# Platelets and Inflammatory Markers in Patients with Gastric Cancer

**DOI:** 10.1155/2013/401623

**Published:** 2013-03-10

**Authors:** Joanna Matowicka-Karna, Zbigniew Kamocki, Beata Polińska, Joanna Osada, Halina Kemona

**Affiliations:** ^1^Department of Clinical Laboratory Diagnostics, Medical University of Bialystok, J. Waszyngton 15a, 15-269 Bialystok, Poland; ^2^Second Department of General and Gastroenterological Surgery, Medical University of Bialystok, M. Sklodowska-Curie 24a, 15-264 Bialystok, Poland; ^3^Department of Haematological Diagnostics, Medical University of Bialystok, J. Waszyngton 15a, 15-269 Bialystok, Poland

## Abstract

The aim of the study was to assess the contribution of platelets and inflammatory markers in gastric cancer. We studied 50 patients. Taking into consideration the advancement of gastric cancer, patients were divided into 3 groups. Group (E)—13 patients with early gastric cancer, group (A)—18 patients with regionally advanced cancer, and group (M)—19 patients with metastatic cancer. The determinations were performed twice prior to surgery and after surgery. In patients with gastric cancer, there is an increase in IL-6 and IL-23 compared with the healthy group. The highest values of IL-6 were obtained in early cancer (more than 8-fold increase), which seems to confirm the presence of acute inflammation. The lowest value of both of these cytokines was obtained in patients with metastatic cancer. In all patients, regardless of tumor stage, there was an increase in the concentration of CRP. An increase of PLT, higher proportion of the percentage of large platelets (LPLT), and increased mean platelet volume (MPV) were observed in the process of disease development. A positive correlation between MPV and LPLT and the accompanying decrease in the concentration of proinflammatory cytokines indicates the presence of an existing relationship between the platelet morphological parameters and the inflammation process in the development of gastric cancer.

## 1. Introduction

Gastric cancer is the second most common cause of deaths from cancer worldwide, and most cases of this type of cancer are found in Japan, China, and Russia [1]. The epidemiology of gastric cancer requires consideration of its two histological subtypes; the more differentiated intestinal type with cells forming glands and the diffuse type with infiltrating and less-differentiated tumor cells, which has a poor prognosis. The intestinal type occurs mainly in areas with a high prevalence of gastric cancer, and its occurrence depends on factors such as sex, diet, smoking, *Helicobacter pylori* infection, atrophic gastritis, chronic gastritis, or intestinal metaplasia [1, 2].

Gastric cancer is usually located in the pyloric antrum and in the pylorus, but in 25% of cases in the body (corpus) and fundus of the stomach. Clinical symptoms of gastric cancer are often nonspecific, and therefore it is diagnosed only in the advanced stage [2]. Generally cancer cells are a source of inflammatory cytokines and growth factors (IL-1*β*, IL-3, IL-6, IL-11, IL-23, and TNF-*α*) [3, 4]. Persistence of the inflammatory process within the tumor leads to an increase in proliferation of tumor cells, angiogenesis, and the inhibition of apoptosis. VEGF accelerates the formation of blood vessels in the tumor, and matrix metalloproteinases (MMPs) facilitate the infiltration and spread to adjacent tissues, which in turn promotes the formation of metastases [3, 5, 6]. 

IL-6 and IL-23, among others, are actively involved in the pathogenesis and development of tumors. They facilitate tumor growth by inhibiting apoptosis of tumor cells and by angiogenesis induction within the tumor [7]. IL-6 is a pleiotropic cytokine involved in immune responses, inflammatory reactions, and haematopoiesis. It may facilitate the destruction of cancer cells by stimulating the activity of antitumor macrophages and by neutrophils apoptosis. IL-6 is one of the elements linking inflammation and angiogenesis in the course of cancers [8, 9]. However, IL-23 strongly induces the secretion of IFN-*γ*, affects the haematopoiesis by stimulating thrombocytopoiesis, and the production of neutrophils, and stimulates the production of acute phase proteins [10, 11]. 

Platelets take part in inflammation and in cancerous diseases. They play an active role in the inflammatory process due to secreted proinflammatory factors, chemokines, and growth factors (such as TXA2, PAF, PF4, IL-1*β*, RANTES, MCP-1, MIP-1*α*, TNF-*α*, VEGF, and PDGF). The tumor cells show a prothrombotic effect by stimulating the aggregation of platelets by agonists (ADP, thrombin). After platelet activation, P-selectin present on the platelets surface is bound to CD24 ligand found on the surface of cancer cells, causing their adherence to endothelial cells. P-selectin participates in both the pathogenesis of thrombosis, as well as in the inflammatory processes in cancer patients. An excessive number of platelets is the cause of a significant increase in the risk of metastases in each stage of cancer and an indicator of poor prognosis, for example, in gastric, lung, and kidney cancer. In cancer disease, an increase in the percentage of large platelets (LPLT) is observed, and because young, metabolically active platelets appear in the circulation, this may lead to an increase in MPV (mean platelet volume) [12, 13]. 

The aim of this study was to find an answer to the question of whether the inflammatory process present in the course of gastric cancer, as well as surgery, affects the number of platelets and their morphological parameters. CRP, IL-6, and IL-23 were the markers used in the assessment of inflammation in patients with gastric cancer. 

## 2. Materials and Methods

The study included 50 patients with gastric cancer treated at the Second Department of General and Gastroenterological Surgery of the Medical University of Bialystok. The group included 35 men and 15 women aged from 31 to 86 years (mean age 65.4 years). The microscopic examination of material obtained during biopsy and/or surgery was used in the clinical diagnosis of gastric cancer patients. The staging of cancer was based on routine histopathological analysis and clinical assessment according to TNM (tumor-nodulus-metastases) classification. Taking into consideration the stage of gastric cancer, patients were divided into 3 groups [14]. Group I (E) included 13 patients (7 M, 6 F) with early stage of gastric cancer. Group II (A) included 18 patients (13 M, 5 F) with locally advanced stage of cancer. Group III (M) included 19 patients (15 M, 4 F), with cancer metastasis (Table 1). In the assessment of the severity of gastric cancer according to UICC classification in group E, 10 patients with stage I and 3 patients with stage II were isolated. In group A, 7 patients with stage IIIA and 11 patients with stage IIIB were isolated, and in group M, 19 patients were classified as stage IV. All patients were qualified for surgical treatment. Tests were carried out twice, before surgery (E1, A1, M1) and 14–16 days after the surgery (E2, A2, M2). The control group (C) comprised of 40 healthy subjects (aged 20–45 years), including 22 men and 18 women.

In accordance with the principles of the *Guidelines for Good Clinical Practice*, all patients gave their consent to the test. The study was approved by the Local Ethics Committee. Venous blood collected on the clot provided the study material.

Determination of morphological parameters of blood platelets 2 mL of venous blood was collected into tubes containing K_2_EDTA (1.5 mg per mL of blood). The assessment of platelet count and their morphological parameters was performed using the ADVIA 2120 hematology analyzer (Siemens). 

The following parameters were determined:

- PLT—platelet count per unit volume of blood (150–350 × 10^9^/L),
- MPV—mean platelet volume calculated on the basis of distribution volume (7,8–11,5 fl),
- LPLT—percentage of large platelets with a volume above 20 fl (0,2–6,0%).

Determination of the concentrations of interleukins 6 and 23 (IL-6 and IL-23) and CRP was performed using ELISA human Quantikine kit (R & D Systems, USA).

## 3. Statistical Analysis

Within the treatment groups and the control group, a statistical analysis of all parameters including age and gender was carried out. The results were statistically analyzed using STATISTICA 8.0. Student's *t*-test was used for determining CRP characteristics consistent with normal distribution, which were evaluated using the Kolmogorov compliance test. In case of PLT, MPV, LPLT, IL-6, and IL-23 determination, the Mann-Whitney test and the Wilcoxon pair test were used for determining characteristics not compliant with normal distribution. The relationships between the measured characteristics of a continuous type were determined using Pearson's correlation coefficient. 

## 4. Results

Platelet count (PLT) in patients with early gastric cancer (E1) and in patients with locally advanced cancer (A1) does not differ significantly in comparison with the values obtained in healthy subjects. However, statistically significant differences were observed in these groups when comparing the results after surgery relative to preoperative values (*P* < 0.05). In patients with metastatic cancer (M1), an increase in platelet count was observed compared with the control group, and this difference is statistically significant. Surgery slightly increased the platelet count in this group (Table 2).

The value of MPV in the study groups (E, A, and M) both before and after surgery did not differ significantly. Only the increase in MPV in patients with advanced cancer (A1) differed statistically significantly compared with the control group (Table 2). 

 The comparison of the LPLT values before and after surgery showed that only in patients with advanced cancer (A), there is a statistically significant difference (*P* < 0.05). LPLT values obtained in group A1 and M1 differ significantly compared with the control group (Table 2). Significantly higher IL-6 concentrations were shown in groups (E1, A1, and M1) before surgery when compared with the control group, and these differences were statistically significant (Table 3).

An increase of IL-23 concentrations was observed in patients in groups E1 and A1 compared with the control group, and the differences were statistically significant. Statistically significant differences between the concentrations of IL-23 were obtained in patients with early cancer before and after surgery (E1 and E2) (Table 3). 

There was a negative correlation between platelet count and IL-23 in group E1 (*r* = −0.8197) and between the percentage of large platelets (LPLT) and IL-6 in group M1 (*r* = −0.5212). There was a positive correlation only between MPV and LPLT in groups E1 (*r* = 0.8804) and M1 (*r* = 0.5971). The comparison of other parameters did not show the existence of any correlation between MPV and LPLT. 

All patients with gastric cancer whose concentration of C-reactive protein value exceeded 10 mg/L were qualified for the study.

## 5. Discussion

Approximately 60% of patients treated for gastric cancer have malnutrition. The result of slowly developing anorexia and the progressive release of cytokines produced by the tumor is protein-energy, vitamin, and trace elements deficiency. One of the first functions to become impaired is that of the immune system. In the course of gastric cancer, a concomitant impairment of the immune mechanisms and the presence of inflammation are observed. Inflammatory mediators (released cytokines, enzymes, and transcription factors) tend to inhibit apoptosis, initiate the development of cancer cells, and enhance their proliferation. Chronic inflammation of the stomach caused by *Helicobacter pylori* often leads to neoplastic transformation. Tumors on the other hand induce inflammation which is the body's immune response [15]. Platelets initiate and facilitate the development of the inflammatory process. They, by adhesion and then activation, together with the cells of the immune system participate in the secretion of chemokines, cytokines, proteases, and procoagulants. Fibrinogen, fibronectin, vitronectin, and vWf contribute to the strengthening of platelet-endothelial adhesion by forming links between GPIIb-IIIa and integrin *α*_*v*_ *β*_3_ or ICAM-1 [16, 17]. 

Neoplasms are usually accompanied by thrombocytosis. If thrombocytopenia occurs in the development of cancer, the cause of this may be the consumption of platelets due to disseminated intravascular coagulation (DIC), which is frequently observed in pancreatic cancer. Thrombocytosis, which may cause an increased risk of metastasis, was observed in gastric, colorectal, lung, kidney, prostate, and reproductive system cancers [3]. Because the tumor can be a source or a stimulator of cytokines such as interleukin, IFN-*γ*, and TNF, this in effect leads to the stimulation of thrombocytopoiesis. In the course of gastric cancer, it is GM-CSF or Tpo through IL-6 which influences the increase in the number of platelets [13, 18, 19]. 

In our study of patients with gastric cancer, inflammation was observed which was subsequently confirmed by the results of determinations of C-reactive protein and IL-6. In patients diagnosed with an early stage of gastric cancer, both the number of platelets and their volume were comparable with values obtained in the control group, whereas in the other study groups (A1 and M1), both the number of platelets and their volume increased with the development of cancer. An increase in platelet count was observed in all groups of patients after surgery. That is probably the result of both the surgical procedure and the simultaneous removal of the spleen carried out during a total gastrectomy. Folman et al. [18] described thrombocytopoiesis agitation and the presence of platelet activation between 7 and 20 days after surgery. In this period, active young platelets appeared in circulation. They possessed more granules and large energy reserves and also greater metabolic activity. 

In our studies, an increase in the percentage of large platelets (LPLT) was observed in both the A1 and M1 groups. Surgical procedure caused 2-field increase of LPLT values in E2 and A2 groups and a slight increase of LPLT in M2 group. The increase of the number of platelets, their volume (MPV), and the percentage of LPLT may confirm stimulation of thrombocytopoiesis in the course of gastric cancer. According to Folman et al. [18], thrombopoietin and IL-6 (both strong stimulators of thrombocytopoiesis) increase the production of platelets and as a result an increase in their number [18]. The gradual increase in the number of platelets in groups E1 to M1 is inversely proportional to the concentration of IL-6. It appears that not only IL-6 is responsible for the stimulation of thrombopoiesis.

The increase of values of MPV and LPLT observed indicates the presence of a subpopulation of young, more active platelets taking part in the process of homeostasis. The presence of a positive correlation between MPV and LPLT in patients with early gastric cancer and in patients with metastatic cancer may indicate the existing relation of morphological parameters and inflammation in cancer development. In spite of the fact that control blood tests were performed two weeks after surgery, such a short period of time may mean that results obtained could contain an error. Loss of blood, metabolic acidosis, and stress connected with surgery can be the reason for activation of compensatory processes. Subsequent stimulation of thrombocytopoiesis causes an increase in the number of platelets and slightly increased values of MPV and LPLT.

In early cancer, the lowest values in platelet count, MPV, and proportion of LPLT were observed. In advanced cancer with metastases to the lymph nodes, all these parameters reached the highest values, which seems what can confirm the maximum level of thrombopoiesis stimulation and platelet activation. Platelet count, MPV, and LPLT increase with the growth of tumor reaching highest values in patients with advanced cancer with metastases to the lymph nodes. A developing tumor promotes stimulation of blood platelets and their activation. From the moment the cancer starts to spread, all of the morphological parameters of blood platelets are reduced, and the falling value of MPV may indicate that intravascular platelet activation occurs. 

According to Park et al. [20], statistically significantly lower values of MPV in patients with gastric cancer are due to degranulation and release of the contents of granules after platelet activation. In Osada et al. [21], of patients with gastric cancer, there were no statistically significant differences when comparing platelet counts in both groups with the control group. MPV and LPLT in all groups of patients were significantly higher than in the control group, whereas MPC in all groups of patients was significantly slighter than in the control group. Osada et al. showed that the number of CD62P antigens on the platelet surface after activation of TRAP (Thrombin Receptor Activation Peptide) in patients with gastric cancer increased by a factor of 6 to 12 compared to a 3-fold increase in healthy subjects. A large number of glycoproteins on the surface of platelets observed in the course of gastric cancer after platelet activation *in vitro* may indicate prothrombotic tendencies. Although it does not change the number of platelets, they exhibit a higher metabolic activity and higher reactivity and as such greater activation capacity [21]. 

In our study, it was found that in all patients with gastric cancer the level of IL-6 was increased in comparison to its values obtained in the control group. In our study, it was found that in all patients with gastric cancer, there is an increase of IL-6, and the reference point was the group of healthy subjects. The highest values of IL-6 were obtained in patients with early cancer (more than 8-fold increase), which seems to confirm the presence of acute inflammation in these patients. Along with the progression of the disease, there was observed a decrease in the production of proinflammatory cytokines, and the lowest IL-6 values were obtained in patients with cancer metastases (more than 3-fold increase). In all patients with gastric cancer regardless of tumor stage, there was an increase in the concentration of C-reactive protein. 

According to Haabeth et al. [22], the presence of active inflammation protects against development of cancer, while a chronic inflammation promotes development of neoplastic diseases. If E1, A1, and M1 groups are compared, it turns out that with the development of cancerous disease, lengthening its duration, and advancement stage, IL-6 concentration is reduced. According to Wu et al. [9], in patients with gastric cancer, IL-6 concentration correlated with the stage of disease and increased where gastric cancer reoccurred. The importance of IL-6 in gastric cancer with invasion of cancer cells to the lymph nodes and distant metastasis formation is also described by Ashizawa et al. [7]. Pyrogen action of this proinflammatory cytokine makes it responsible for the phenomenon of cachexia, occurrence of fever, weight loss, and other debilitating symptoms of tumor progression. Serum IL-6 also increases in chronic inflammations, including viral diseases and bacterial infections, autoimmune diseases, ischemia, and diabetes. The increase in blood levels of IL-6 observed was accompanied by elevated concentrations of CRP; therefore, determining the concentration of this cytokine was supposed to be a predictor of survival in patients with gastric cancer, and determining the concentration of CRP was to be a factor in the progression of cancer and stages of malignancy [7, 23]. Also according to Kim et al. [4], identifying IL-6 and CRP is dependant on the stage of cancer and they may be markers of tumor invasion. 

Markers of inflammation may also include IL-23, which stimulates the production of platelets and strongly induces the production of acute phase proteins. IL-23 promotes an inflammatory response and enhances angiogenesis, and its expression is particularly elevated in solid tumors [10, 24]. In this study, their highest levels of IL-23, like IL-6, were obtained in patients with early cancer, while the lowest value of both these cytokines was obtained in patients with disseminated cancer. It seems that the stimulation of production of proinflammatory cytokines such as IL-6 and IL-23 is highest when gastric cancer is still developing. The progression of the disease and metastatic of the tumor is followed by gradual decrease in the production of both cytokines. 

The advancement stage of gastric cancer, which is correlated with a decrease in IL-6 and IL-23 levels does not seem to bear any relation to the number of platelets or their morphological parameters. Observed thrombopoiesis stimulation in A1 and M1 groups (when compared to E1 group) is not directly dependent on IL-6 and IL-23. There may be other factors that with the development of malignancy stimulate thrombopoiesis process.

The high rates of gastric cancer mortality may be related to direct invasion into the adjacent organs, lymph node metastasis, and distant metastasis of gastric cancer. IL-6 plays a positive role as a prognostic factor in lymph node metastasis and advanced gastric cancer [7]. 

NF-kappaB is a potential sign for inflammation; NF-kappaB has been associated with the progression of the disease in various types of cancer. NF-kappaB is a potential sign for inflammation; NF-kappaB has been associated with the progression of the disease in various types of cancer. NF-kappaB is a factor, which is attribute to the role of inhibitor of apoptosis, and this affects the development of many cancers. However, whether the expression of IL-6 correlates with the expression of NF-kappaB in patients suffering from gastric cancer remains unclear [25]. 

## Figures and Tables

**Table 1:** The stage of gastric cancer.

Group tested		
Tumor stage (TNM classification)	I + II (*n* = 13)	26%
	III (*n* = 18)	36%
	IV (*n* = 19)	38%

Depth of tumor invasion	T1 (*n* = 4)	11%
	T2 (*n* = 7)	19%
	T3 (*n* = 15)	40%
	T4 (*n* = 11)	30%

Lymph node metastases	N0 (*n* = 7)	17%
	N1 (*n* = 4)	9%
	N2 (*n* = 13)	31%
	N3 (*n* = 18)	43%

Distant metastases	M0 (*n* = 9)	47%
	M1 (*n* = 10)	53%

**Table 2:** The mean platelet count and their morphological parameters in patients suffering from gastric cancer—early gastric cancer, E (E1 before surgery, E2 after surgery), advanced cancer with metastasis to lymph nodes, A (A1 before surgery, A2 after surgery), and metastatic tumor, M (M1 before surgery, M2 after surgery) and in normal donors (C).

	PLT	MPV	LPLT
	X ± SD	X ± SD	X ± SD
Study group	248.53 ± 43.92	8.81 ± 0.64	6.1 ± 4.45
E1	E1 : C	E1 : C	E1 : C
*N* = 13	n.s.	n.s.	n.s.

Study group	528.43 ± 326.13	8.92 ± 0.82	14.1 ± 7.61
E2	**E1 : E2**	E1 : E2	E1 : E2
*N* = 13	P < 0.05*	n.s.	n.s.

Study group	344.28 ± 266.40	9.48 ± 0.87	10.17 ± 8.56
A1	A1 : C	**A1 : C**	**A1 : C**
*N* = 18	n.s.	P < 0.05*	P < 0.05*

Study group	584.25 ± 237.54	9.64 ± 0.79	20.83 ± 12.43
A2	**A1 : A2**	A1:A2	**A1 : A2**
*N* = 18	P < 0.05*	n.s.	P < 0.05*

Study group	335.84 ± 154.08	9.04 ± 0.86	8.16 ± 3.47
M1	**M1 : C **	M1 : C	**M1 : C**
*N* = 19	P < 0.01*	n.s.	P < 0.01*

Study group	451.55 ± 210.74	9.23 ± 1.10	10.27 ± 5.69
M2	M1 : M2	M1 : M2	M1 : M2
*N* = 19	n.s.	n.s.	n.s.

Control group			
C	247.78 ± 44.74	8.68 ± 0.64	5.34 ± 1.81
*N* = 40			

*Statistically significant when *P* < 0.05.

**Table 3:** The concentration of IL-6 and IL-23 in patients suffering from gastric cancer—early gastric cancer, E (E1 before surgery, E2 after surgery), advanced cancer with metastasis to lymph nodes, A (A1 before surgery, A2 after surgery), and metastatic tumor, M (M1 before surgery, M2 after surgery) and in normal donors (C).

	IL-6	IL-23
	X ± SD	X ± SD
Study group	21.28 ± 6.34	10.89 ± 5.46
E1	**E1 : C **	**E1 : C **
*N* = 13	P < 0.01*	P < 0.05*

Study group	10.86 ± 8.93	3.88 ± 2.59
E2	E1 : E2	**E1 : E2 **
*N* = 13	n.s.	P<0.01*

Study group	11.51 ± 8.44	9.46 ± 8.81
A1	**A1 : C **	**A1 : C **
*N* = 18	P < 0.001*	P < 0.001*

Study group	20.42 ± 10.68	10.80 ± 8.24
A2	A1 : A2	A1 : A2
*N* = 18	n.s.	n.s.

Study group	8.44 ± 5.63	5.94 ± 4.70
M1	**M1 : C**	M1 : C
*N* = 19	P < 0.001*	n.s.

Study group	15.26 ± 14.73	3.05 ± 2.47
M2	M1 : M2	M1 : M2
*N* = 19	n.s.	n.s.

Control group		
C	2.45 ± 1.44	5.21 ± 3.65
*N* = 40		

*Statistically significant when *P* < 0.05.
